# Multicellular Human Cardiac Organoids Transcriptomically Model Distinct Tissue-Level Features of Adult Myocardium

**DOI:** 10.3390/ijms22168482

**Published:** 2021-08-06

**Authors:** Charles M. Kerr, Dylan Richards, Donald R. Menick, Kristine Y. Deleon-Pennell, Ying Mei

**Affiliations:** 1 Molecular Cell Biology and Pathobiology Program, Medical University of South Carolina, Charleston, SC 29425, USA; kerrch@musc.edu; 2 Immunology Translational Sciences, Janssen Research and Development, LLC, Spring House, PA 19477, USA; dricha14@its.jnj.com; 3 Division of Cardiology, Department of Medicine, Gazes Cardiac Research Institute, Medical University of South Carolina, Charleston, SC 29425, USA; menickd@musc.edu (D.R.M.); deleonky@musc.edu (K.Y.D.-P.); 4 Ralph H. Johnson Veterans Affairs Medical Center, Medical University of South Carolina, Charleston, SC 29401, USA; 5 Bioengineering Department, Clemson University, Clemson, SC 29634, USA; 6 Department of Regenerative Medicine and Cell Biology, Medical University of South Carolina, Charleston, SC 2942, USA

**Keywords:** hiPSC-CMs, human cardiac organoids, transcriptome, engineered heart tissue, human myocardium, 3D culture, cardiac microtissue, RNA-sequencing

## Abstract

Human-induced pluripotent stem cell-derived cardiomyocytes (hiPSC-CMs) have been widely used for disease modeling and drug cardiotoxicity screening. To this end, we recently developed human cardiac organoids (hCOs) for modeling human myocardium. Here, we perform a transcriptomic analysis of various in vitro hiPSC-CM platforms (2D iPSC-CM, 3D iPSC-CM and hCOs) to deduce the strengths and limitations of these in vitro models. We further compared iPSC-CM models to human myocardium samples. Our data show that the 3D in vitro environment of 3D hiPSC-CMs and hCOs stimulates the expression of genes associated with tissue formation. The hCOs demonstrated diverse physiologically relevant cellular functions compared to the hiPSC-CM only models. Including other cardiac cell types within hCOs led to more transcriptomic similarities to adult myocardium. hCOs lack matured cardiomyocytes and immune cells, which limits a complete replication of human adult myocardium. In conclusion, 3D hCOs are transcriptomically similar to myocardium, and future developments of engineered 3D cardiac models would benefit from diversifying cell populations, especially immune cells.

## 1. Introduction

Cardiovascular disease is the leading cause of death worldwide [1]. While interventional therapies have significantly reduced mortality in patients suffering from acute cardiovascular disease, the success of these therapies contributes to a higher prevalence of heart failure in an aging population [2,3]. However, fewer novel cardiovascular therapies succeed through clinical trials [4], which has been attributed to low tolerance in drug toxicity [5], high research and development costs [6], and interspecies differences between animals and humans [7]. These challenges impeding cardiovascular drug development illustrate a growing need for effective in vitro models of human myocardium.

The need for human myocardium models has prompted extensive applications of human induced pluripotent stem cell-derived cardiomyocytes (hiPSC-CM) in a two-dimensional (2D) culture for cardiovascular drug and toxicity screening [8,9,10]. While 2D platforms provide effective cardiomyocyte models to screen drug cardiotoxicities, they provide an inadequate representation of human heart tissue; they lack a multicellular, 3D environment for mimicking the diverse and intricate collaboration of cardiac cell types inherent to heart tissue. To address the inadequacy of 2D monocellular screening platforms, our lab has developed an in vitro human cardiac organoid (hCO) model consisting of hiPSC-CMs, primary adult cardiac fibroblasts, primary endothelial cells and stromal cells to recapitulate human myocardium [11]. We recently demonstrated that our hCOs recapitulated tissue-level hallmarks of myocardial infarction and reproduced process-level transcriptomic shifts occurring in both human ischemic cardiomyopathy and a murine myocardial infarction [12]. Here, we aim to evaluate our hCO’s recapitulation of homeostatic human myocardium and characterize the biomimetic transcriptomic properties of 3D cardiac microtissues.

In this study, we investigated the transcriptomes of in vitro human cardiovascular models (2D iPSC-CMs, 3D iPSC-CMs and hCOs) and compared them to publicly available bulk RNA-seq datasets of healthy human myocardium derived from fetal atria, fetal ventricles and adult ventricles. Our data show that a 3D in vitro environment stimulates the expression of tissue formation genes, such as those related to angiogenesis, extracellular matrix regulation and cell communication. We further demonstrate that hCOs best recapitulate cellular diversity and share the highest transcriptomic similarity to human myocardium, when compared to 3D hiPSC-CMs and 2D hiPSC-CMs. When compared to human myocardium, hCOs exhibit a distinct transcriptomic signature that is similar to fetal myocardium and adult myocardium without an immune cell population. The lack of additional cell types (e.g., immune cells), limits the ability of hCOs to fully recapitulate human adult myocardium, suggesting an area of further research. Overall, this study demonstrates that hCOs mimic the 3D characteristics and cellular composition of human myocardium.

## 2. Results

### 2.1. Analysis of In Vitro Cardiovascular Models

#### 2.1.1. Principal Component Analysis (PCA) and Gene Set Enrichment Analysis (GSEA) of In Vitro Cardiac Models

**Figure 1:**
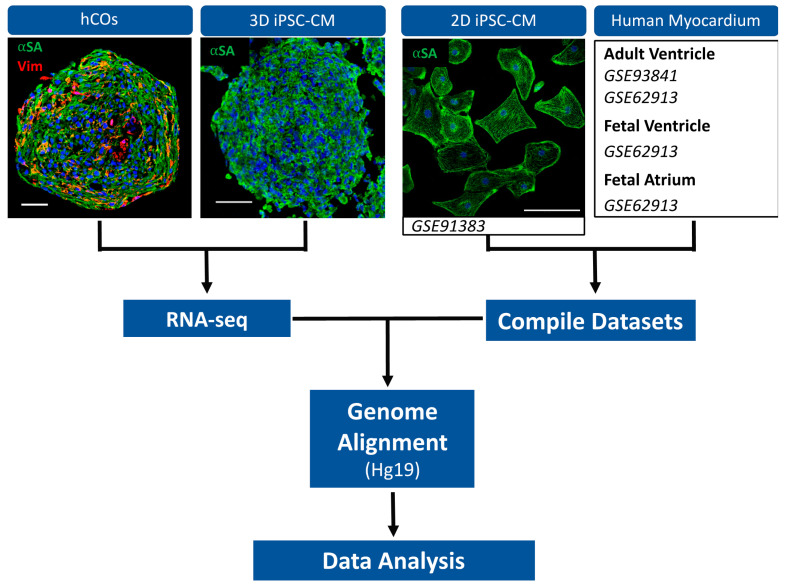
Schematic representation of RNA-seq workflow. SA (alpha sarcomeric actinin; hiPSC-CM marker). Vim (vimentin; fibroblast marker). Scale bars are 50μm.

We have previously demonstrated the unique benefit of including supporting cell types (e.g., adult cardiac fibroblasts, endothelial cells and stromal cells) within 3D cardiac models for functional modeling of cardiovascular disease [12]. This prompted us to further investigate the benefits of incorporating multiple cell types within hCOs compared to 2D and 3D hiPSC-CMs monocellular models. To this end, we compiled a dataset of publicly available RNA-seq data including 2D hiPSC-CMs (*n* = 3, GSE91383 [13]) and our 3D hCOs (*n* = 3, GSE113871 [12]). In addition, we performed RNA-seq on spherical hiPSC-CMs (3D hiPSC-CMs) (*n* = 3), fabricated using our previously established 3D culture method [14]. The hiPSC-CMs in all in vitro models were donor matched and purchased from the same supplier (Fujifilm) to reduce the iPSC-CM differentiation variability. All the in vitro groups were cultured for similar durations prior to RNA isolation: a 14-day culture for the 3D hiPSC-CMs and the hCOs and a 12-day culture for the 2D hiPSC-CMs. We assume the 2 days additional culture of the 12-day-old 2D hiPSC-CMs will not significantly affect their transcriptome. An outline of our RNA-seq experimental design is shown in Figure 1.

**Figure 2:**
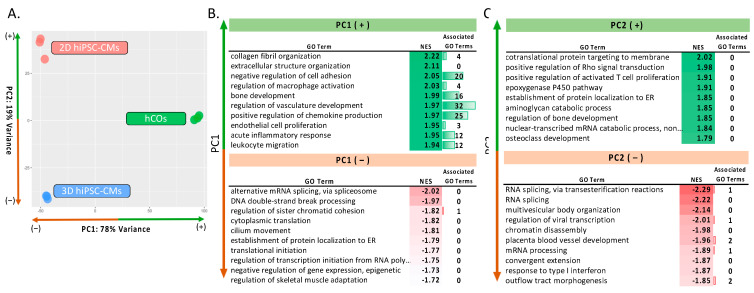
PCA and GSEA analysis of in vitro cardiac models. GSEA analysis performed using gene loadings from PC1 and PC2. Associated GO terms depict redundant GO terms filtered out through REViGO. (**A**) PCA of 2D hiPSC-CMs, 3D hiPSC-CMs and hCOs. (**B**) 10 significantly enriched GO terms along PC1(+)(top) and PC1(–)(bottom): PC1(+) depicted pathways enriched in hCOs; PC1(–) depicts pathways co-enriched in 2D hiPSC-CMs and 3D hiPSC-CMs. (**C**) 10 significantly enriched GO terms along PC2(+)(top) and PC2(–)(bottom): PC2(+) represents pathways enriched in 2D hiPSC-CMs; PC2(–) represents pathways enriched in 3D hiPSC-CMs.

We initially investigated the global transcriptomic differences between human 2D hiPSC-CMs, 3D hiPSC-CMs and hCOs using a Principal Component Analysis (PCA). The analysis revealed that each in vitro culture method demonstrated a unique transcriptomic signature across the PC1 and PC2 axis, Figure 2A. The 2D hiPSC-CMs and the 3D hiPSC-CMs grouped similarly along PC1, representing 78% of the sample variation, and separate from hCOs, reflecting the differences in cellular composition between the hCOs and the monocellular models. In contrast, PC2, representing 19% of the sample variation, best captures the differences between the 2D hiPSC-CMs and the 3D hiPSC-CMs; the hCOs fell between the two groups indicating a similarity to both groups along this axis.

Gene set enrichment analysis (GSEA) was performed on each axis using the PCA gene loadings, as previously described [12]. Enriched pathways were further filtered using REViGO to remove the redundant gene ontology (GO) terms [15], noted as associated GO terms. Numerous GO terms were found within each analysis; the most pertinent, significant and enriched terms are shown in Figure 2. A full list of GO terms can be found in Table S1. The PC1(+), representative of the organoid group, showed significant enrichment in “collagen fibril organization” and “extracellular structure organization”, Figure 2B. Since fibroblasts’ central role in myocardium encompasses extracellular matrix (ECM) homeostasis and pathological ECM remodeling [16], these data support the concept that including fibroblasts within hCOs leads to enhanced ECM functionality. Similarly, “bone development” showed enrichment, which includes COL1A1 expression, supporting the ECM contributions of fibroblasts. “Regulation of vasculature development” was enriched with 32 associated GO terms, demonstrating an enhanced vascularization capacity and is supported by the enrichment of “endothelial cell proliferation” with three associated GO terms. Endothelial cells serve a vital role in cardiac remodeling following pathological stress [17]; their inclusion within hCOs may be necessary to capture tissue-level responses to stress. Interestingly, several terms related to immunological function (e.g., “acute inflammatory response”, “positive regulation of chemokine production”, “regulation of macrophage activation” and “leukocyte migration”) were also enriched in PC1(+), indicating immunoregulatory functions. While our model lacks immune cells, a recent study demonstrated transcriptomic differences in immune cell trafficking (e.g., Il-6 signaling) between fetal and adult cardiac fibroblast in non-pathogenic conditions demonstrating a homeostatic immunoregulatory function [18]. Further, both fibroblasts and endothelial cells have been shown to play immunoregulatory roles in heart failure [19].

Meanwhile, PC1(–), representative of the monocellular models, showed enriched pathways in regulating gene expression (e.g., “alternative mRNA splicing”, “translation initiation” and “negative regulation of gene expression (epigenetically)”), Figure 2B. “Skeletal muscle adaptation”, which includes essential cardiomyocyte genes (e.g., ATP2A2, TNNC1 and MYH7) was enriched within monocellular 2D/3D hiPSC-CM. This could be attributed to the following different cell compositions within the models: 100% hiPSC-CMs in 2D/3D hiPSC-CMs and 50% hiPSC-CMs in hCOs. Overall, these data support the concept that the incorporation of multiple cell types within the hCOs shows more diverse myocardial characteristics regarding ECM dynamics, vascularization and early immunological responses.

In Figure 2C, PC2(+) represents unique transcriptomic differences between 2D hiPSC-CMs and 3D hiPSC-CMs. GSEA analysis revealed that several pathways enriched in 2D hiPSC-CMs were related to “protein translation”. Previous reports suggest cell culture substrates’ stiffness (e.g., tissue culture plates) stimulate cardiomyocyte hypertrophic pathways [20]. This is supported by the enrichment of the “positive regulation of Rho protein signal transduction” in 2D hiPSC-CMs, which plays a role in mechanical-stress-induced cardiomyocyte hypertrophy [21]. On the other hand, “RNA splicing” was enriched in PC2(–), indicating changes in the regulation of transcription. We speculate that these changes reflect the adaptation of hiPSC-CM to a 3D environment, having previously been differentiated and cultured as a 2D monolayer. Notably, “placental blood vessel development” was enriched in PC2(–). This is supported by our previous studies that show the existence of hypoxic gradients within our 3D hiPSC-CMs and hCOs [12,22]. In summary, our data demonstrate 2D hiPSC-CM stimulated hypertrophic growth compared to 3D hiPSC-CM yet lacks more complex tissue-level signatures (e.g., ECM dynamics, vascularization and diffuse gradients).

#### 2.1.2. Differential Gene Expression (DGE) Analysis of 3D Cardiac Models

**Figure 3:**
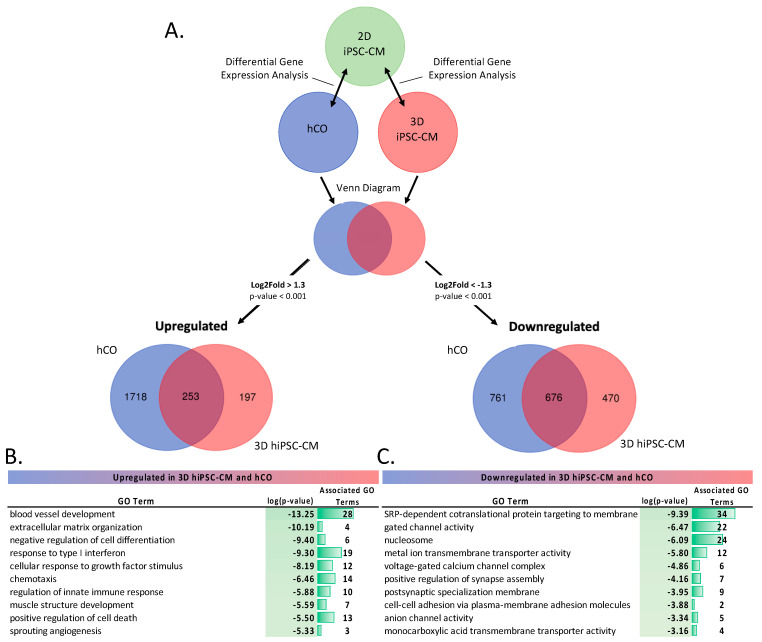
DGE analysis of in vitro models (**A**) Schematic representation of the DGE analysis. DESeq2 was performed on hCOs (*n* = 3) and 3D hiPSC-CMs (*n* = 3) samples compared to 2D hiPSC-CMs (*n* = 3) samples. Results were filtered for DEGs with a |log2fold change| > 1.3 and *p*-value < 0.001. Differentially expressed genes were split into upregulated (log2fold change > 1.3) and downregulated (log2foldchange < −1.3). A Venn diagram of upregulated and downregulated genes was performed. (**B**,**C**) Pathway analysis of upregulated (**B**) and downregulated (**C**) differentially expressed genes expressed in both hCOs and 3D hiPSC-CMs.

We next performed differential gene expression (DEG) analysis to investigate the impact of 3D culture conditions. We first compared the DEGs between (1) hCOs versus 2D hiPSC-CMs and (2) 3D versus 2D hiPSC-CMs, Figure 3A. Then, we isolated the upregulated and downregulated genes in both DEG comparisons. Using a Venn diagram, we identified the unique and similarly expressed genes within the hCOs and 3D hiPSC-CMs in relation to the 2D hiPSC-CMs. A full list of genes can be found in Table S2. Pathway analysis was performed on the upregulated and downregulated genes using Metascape [23]. There were 253 genes upregulated in both the 3D hiPSC-CMs and the hCOs. “Blood vessel development” was the most significantly enriched and had the most associated GO terms. These data suggest that the 3D environment promotes blood vessel development. Further, this promotion is independent of endothelial cells, due to their exclusion in the 3D hiPSC-CMs group. Interestingly, several terms related to immune response were significantly upregulated in the 3D models. “Positive regulation of cell death” along with 13 associated GO terms were also enriched, suggesting that perhaps dying cells (e.g., from hypoxic stress) stimulate the expression of genes involved in the initial activation of the immune system. “Muscle structure development” was also upregulated, suggesting the 3D environment enhanced the development of cardiac tissue. Of the 676 genes that were downregulated in both the 3D hiPSC-CMs and the hCOs, several ion channel-related terms were enriched (e.g., “voltage gated calcium channel complex”, “anion channel activity”), indicating that the 2D hiPSC-CMs enhances ion channel development, more than the 3D hiPSC-CMs and the hCOs. Further, the enrichment of “cell–cell adhesion” within the 2D hiPSC-CMs highlights unique cell–cell interactions, which may further enhance ion channel gene expression. Thus, our data indicate that 3D in vitro conditions enhanced vascularization and ECM properties, while 2D hiPSC-CMs can promote electrophysiological cardiomyocyte properties.

### 2.2. Comparison of In Vitro Cardiac Models and Human Myocardium

#### 2.2.1. PCA and GSEA of In Vitro Cardiac Models and Human Myocardium

**Figure 4:**
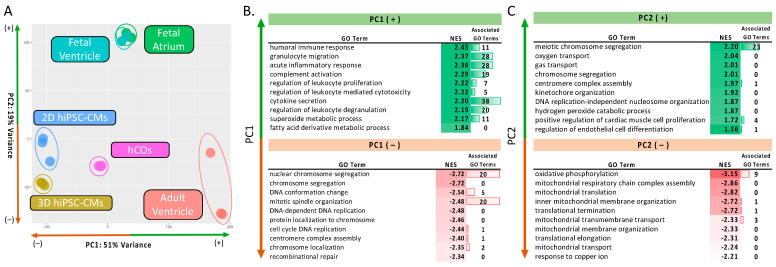
PCA and GSEA analysis of in vitro and human myocardium samples (**A**) PCA analysis of 2D hiPSC-CMs (*n* = 3), 3D hiPSC-CMs (*n* = 3), hCOs (*n* = 3), fetal atrium (*n* = 2), fetal ventricle (*n* = 2) and adult ventricle (*n* = 3) samples. (**B**) GSEA analysis performed using the gene loadings from PC1. Data show 10 significantly enriched GO terms. Associated GO terms depict redundant GO terms removed through REViGO analysis. PC1(+) (top) depicted pathways enriched in adult ventricles; PC1(–) (bottom) depicts pathways enriched in in vitro monocellular iPSC-CM c (**C**) 10 significant enriched GO terms after applying GSEA to PC2. PC2(+) (top) represents pathways enriched in fetal myocardium; PC2(–) (bottom) represents pathways enriched in non-fetal myocardium samples.

While our in vitro analysis demonstrated a more diverse cellular function within hCOs, reflecting its multicellular composition, we sought to identify the accuracy of hCOs resembling human myocardium. We combined our in vitro data set with human myocardium data: human fetal ventricle (*n* = 2; GSE62913 [24]), human fetal atrium (*n* = 2; GSE62913 [24]) and human adult ventricle (*n* = 3; GSE93841 [25], GSE62913 [24]). In the PCA of our in vitro and myocardium samples, Figure 4A, PC1 represented 51% of the sample variation. Along PC1, the hCOs grouped closest to fetal myocardium yet closer to adult ventricles compared to any other in vitro condition. GSEA was performed, repeating the protocol used in Figure 2. A full list of enriched GO terms for PC1 and PC2 can be found in Table S3. PC1 revealed that the genes responsible for PC1(+) corresponded to immune activation and regulation, Figure 4B. While this highlights the lack of immune cell populations within all the in vitro models, it also indicates a high similarity between hCOs and native cardiac cell types of adult myocardium. PC1(–) showed high enrichment for terms related to cell cycle and DNA replication. These data suggest that the monocellular nature of the 2D hiPSC-CMs and 3D hiPSC-CMs groups exhibit increased nuclear activity. While the hiPSC-CMs have limited proliferative capabilities, the enriched active nuclear terms (e.g., cell cycle DNA replication) alludes to nuclear division, suggesting active binucleation and proliferation. Binucleation does occur during early stages of cardiomyocyte development and indicates maturation [26]. While no mitotic pathways were enriched, Voges et al. [27] reported hiPSC-CM proliferation in their hCOs injury model suggesting that proliferation could also be occurring. Notably, the fetal myocardium and hCOs centered along the PC1 axis, demonstrating that they share traits of nucleus division and immunoregulatory function. This is supported by the fact that fetal tissues contain developed innate immune cell activity while lacking matured, adaptive immunity [28].

Along PC2, fetal myocardium was isolated from the other groups. PC2(–) demonstrated the enrichment of terms associated with metabolic demand (e.g., “oxygen transport”), Figure 4C. This may reflect the hypoxic prenatal environment and oxygen independent metabolism of fetal myocardium [29]. In contrast, the hCOs were fabricated with adult human cardiac fibroblasts, endothelial cells and stromal cells and were maintained in normoxia (18% O_2_). Interestingly, PC2(+) was dictated largely by genes enriched in meiotic cells cycle. Since meiosis is limited to gametes, we presume this term is upregulated due to enhanced nuclear activity. Additionally, other nuclear segregation terms (e.g., “centromere complex assembly”) were also enriched supporting this presumption. In summary, our data indicated that hCOs have a distinct transcriptomic signature resembling fetal myocardium in normoxic culture conditions and adult myocardium without an immune cell population.

#### 2.2.2. Gene Set Variation Analysis (GSVA) of In Vitro Cardiac Models and Human Myocardium

**Figure 5:**
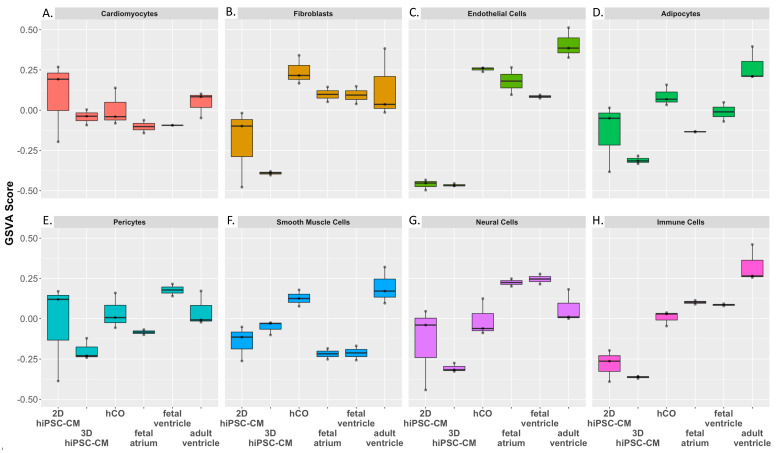
GSVA of in vitro models and human myocardium. GSVA was performed using cell-type specific gene sets from the Heart Cell Atlas. The following self-defined cell-type specific gene sets were created: (**A**) Cardiomyocytes, (**B**) Fibroblasts, (**C**) Endothelial cells, (**D**) Pericytes, (**E**) Smooth Muscle Cells, (**F**) Neural Cells, (**G**), Adipocytes and (**H**) Immune Cells.

Since hCOs contain multiple cell types, we sought to assess whether hCOs accurately recapitulate the transcriptome of corresponding cell types in human myocardium. We extracted the data of cell-type specific genes from the human Heart Cell Atlas [30], a large single-cell and single-nuclei RNA-sequencing dataset from 6 regions of 14 healthy human hearts. Using these cell-type specific gene sets, we constructed a simplified dataset of the top uniquely expressed genes for adipocytes, cardiomyocytes, endothelial cells, immune cells, neural cells, pericytes, smooth muscle cells and fibroblasts. We then performed a gene set variation analysis (GSVA) using highly expressed genes within each cell-type specific gene set (signature). A full gene list per cell-type can be found in Table S4. In our analysis, both 3D hPSC-CMs and hCOs have gene enrichment (GSVA) scores like that of fetal ventricle and adult ventricles, Figure 5A. On the other hand, 2D hPSC-CMs exhibited an artificial cardiac gene enrichment compared to adult ventricles, consistent with the hypertrophic gene expression of hPSC-CMs on stiff substrates (Figure 2C). Both the 2D hiPSC-CMs and the 3D hiPSC-CMs showed lower enrichment in the fibroblast and endothelial cell signatures, Figure 5C and Figure 5D, respectively, while the hCOs showed the GSVA score resembling adult ventricles. These data support that fibroblast and endothelial cells within the hCOs creates a biological platform that is consistent with adult myocardium. hCOs also demonstrated a GSVA score most similar to adult ventricles within pericyte, smooth muscle cell, neural cell and adipocyte signatures when compared to other in vitro groups, Figure 5E–G, respectively. Additionally, hCOs showed immune cell enrichment similar to fetal atrium and fetal ventricles, Figure 5H, consistent with the result in Figure 4. This further supports our earlier finding that incorporating other cell types within a model creates an enhanced immunoregulatory characteristic, not seen in monocellular hiPSC-CM models.

#### 2.2.3. K-Means Cluster Analysis of Cardiomyocyte Specific Genes within In Vitro Cardiac Models

**Figure 6:**
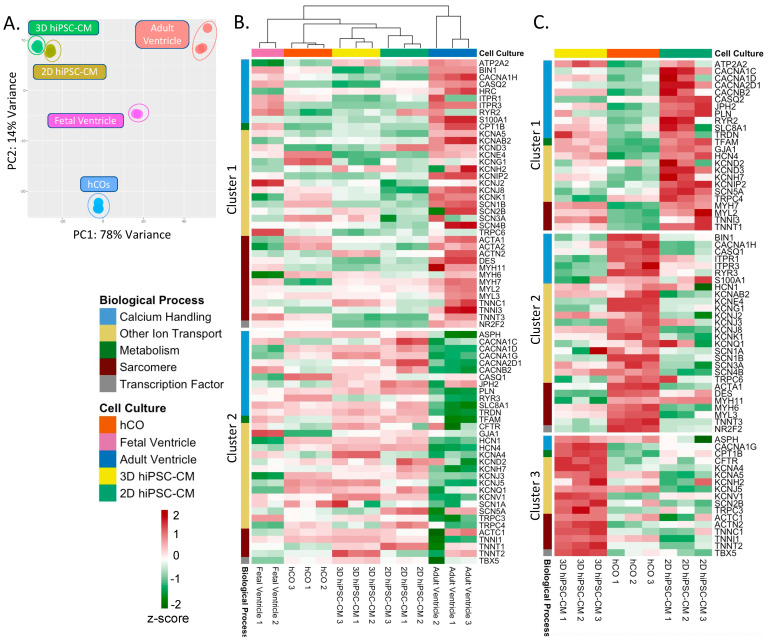
PCA and k-means cluster analysis of a cardiomyocyte specific gene set. (**A**) PCA of cardiomyocyte gene expression in 2D hiPSC-CMs, 3D hiPSC-CMs, hCOs, fetal ventricles and adult ventricles. (**B**) Heatmap of cardiomyocyte gene expression of 2D hiPSC-CMs, 3D hiPSC-CMs, hCOs, fetal ventricles and adult ventricles. Genes were categorized by reported biological processes characteristic of cardiomyocytes. (**C**) Comparison of in vitro hiPSC-CM platforms. Genes were categorized by known cellular functions characteristic of cardiomyocytes.

As hiPSC-CMs are considered to resemble fetal cardiomyocytes, there is widespread emphasis on cardiomyocyte maturation within in vitro models for disease modeling [31,32]. To investigate whether one of our in vitro models enhance cardiomyocyte maturation, we compared the in vitro models to fetal and adult ventricular tissue using a PCA and a k-means cluster analysis using previously reported cardiomyocyte specific genes [33,34,35], Table S5. A PCA revealed hCOs’ expression of cardiomyocyte genes resembled adult ventricles more so than the other in vitro models across PC1; PC1 represented 78% of variance, Figure 6A. This indicates that hCOs can provide a supportive microenvironment to promote biomimetic maturation of hiPSC-CM. Meanwhile, PC2, representing only 14% of the variance, showed that 2D and 3D hiPSC-CMs exhibit adult ventricular qualities not seen in hCOs. K-means cluster analysis revealed the following two clusters of cardiomyocyte genes: high adult ventricular expression in adult myocardium (i.e., cluster 1) and low adult ventricular expression (i.e., cluster 2), Figure 6B. Several cardiomyocyte genes in hCOs showed more similar expression to adult ventricles (e.g., KCNE5, ACTA1, TNNT3, SCN1B) compared to other in vitro methods. Meanwhile, genes in 2D and 3D hiPSC-CMs also showed more resemblance to adult ventricle compared to hCOs (e.g., TNNT2, TNNC1, MYH7, ATP2A2). To further deduce the strengths and weaknesses of each in vitro model in terms of cardiomyocyte function, we replicated the k-means cluster analysis in Figure 6A using only the in vitro models, Figure 6B. We identified the following three main clusters of genes that separated each in vitro group: high 2D hiPSC-CM expression (i.e., cluster 1), high hCOs expression (i.e., cluster 2) and high 2D hiPSC-CM (i.e., cluster 3). BIN1 and DES, essential for sarcoplasmic reticulum Ca-channel development [36] and sarcomere structure development{Brodehl, 2018 #482], respectively, were highly expressed in hCOs. Meanwhile, GJA1 and RYR2 showed higher expression within 2D hiPSC-CMs compared to our in vitro models. However, the amount of expression in 2D hiPSC-CMs was higher than that of adult myocardium. Generally, 3D hiPSC-CMs showed an elevated expression in characteristic sarcomere and contractional proteins (e.g., TNNC1), hCOs showed a higher expression of many potassium channels (e.g., KCNJ2) and 2D hiPSC-CMs demonstrated an enhanced calcium specific channel expression (e.g., CACNA1C). These data suggest that hCOs best replicate adult cardiomyocyte gene expression, while 2D and 3D hiPSC-CMs exhibit certain qualities of adult cardiomyocytes.

## 3. Discussion

Three-dimensional cardiac microtissues hold tremendous promise for cardiovascular drug cardiotoxicity screening and disease modeling, yet they typically lack a diverse cell population. The successful inclusion of other cell types within in vitro models could allude to other cardiotoxicity effects independent of cardiomyocytes. Our hCOs incorporate immature hiPSC-CMs, donor mismatched and primary cardiac fibroblasts and a non-cardiac specific endothelial cell population. We sought to investigate to what extent our 3D cardiac model recapitulates adult myocardium. Knowing the strengths and limitation of our model could further direct future investigations towards a higher-fidelity in vitro myocardium platform.

Here, we first demonstrated the fundamental transcriptomic differences among three in vitro cardiac models. Our analysis shows that 2D hiPSC-CMs have enhanced the transcription of genes related to cardiac conduction and hypertrophic growth, Figure 2C, Figure 3C and Figure 6. Meanwhile, hCOs showed the highest similarity to human adult myocardial transcriptome by demonstrating fibroblast specific ECM organization, endothelial cell vascularization and early immune cell regulation, Figure 2B and Figure 4B. In this regard, hCOs best capture cell-type specific and collaborative interactions between the cell types found within adult myocardium. Independent of the diverse cell populations, the 3D environments of both the 3D hiPSC-CMs and hCOs demonstrated enhanced cell–cell communication, ECM organization and vasculature regulation, Figure 3B. In addition, hCOs demonstrated more resemblance to cardiomyocyte specific genes of fetal and adult myocardium, Figure 6. Other studies have demonstrated enhanced cardiomyocyte maturation due to electrical, mechanical and/or biochemical stimuli in 2D and 3D environments [31,32]. Our data suggest including additional cells type in 3D could enhanced cardiomyocyte specific genes towards a natural maturing cardiomyocyte phenotype, Figure 6. In support of this, Giacomelli et al. [37] reported enhanced cardiomyocyte maturation resulting from a cross talk of hiPSC-CM, and hiPSC-derived cardiac fibroblasts and hiPSC-derived cardiac endothelial cells. Interestingly, our data suggest that monocellular hiPSC-CM models may provide better insight into how a drug candidate affects cardiomyocyte specific responses (e.g., cardiac conduction). For future disease modeling and drug discovery, the combination of human hCOs and hiPSC-CM, only 2D/3D models would provide a comprehensive overview of the diverse responses of human myocardium to drug candidate treatments.

Since our hCOs do not incorporate every cell type found within myocardium, we sought to identify the strengths and weaknesses of our hCOs through a direct comparison to human myocardium transcriptomes. Several groups have reported transcriptomic shifts of their engineered myocardium focusing on adult cardiomyocyte phenotypes [25,37]. Our goal was to compare our organoid model directly to human myocardium to examine the tissue-level characteristics. Our RNA-seq data showed that hCOs displayed a transcriptome more similar to adult myocardium compared to the other in vitro methods and fetal myocardium, Figure 4A. However, there are still deficiencies within hCOs that limit their full recapitulation of adult myocardium. Mainly, our results highlighted the lack of immune cells within our hCOs. To the best of our knowledge, no in vitro cardiac model has incorporated immune cells. Since immune cells have strong regulatory actions involved in cardiovascular disease [38], the incorporation of immune cells could create more comprehensive in vitro disease models and more accurate tissue-level responses to drug candidates. Nonetheless, the identification of suitable cell types (e.g., macrophages, T cells, etc.), culture media and fabrication strategies necessitate additional efforts.

Several human hCOs models are composed of only hiPSC-derived cell types (e.g., cardiomyocytes, cardiac fibroblasts and endothelial cells) [25,37,39,40]. However, Zhang et al. demonstrated that iPSC-derived cardiac fibroblasts are more similar to embryonic cardiac fibroblasts [39]. Additionally, fetal and adult cardiac fibroblasts naturally exhibit unique transcriptomic differences [18] and the age of fibroblasts can also impact cardiomyocyte maturation and development [40,41]. Additionally, recent studies have demonstrated that aged cardiac fibroblasts exhibit transcriptomic heterogeneity among fibroblast sub-populations [42] and contribute to declined heart function [42]. These reports suggest that our hCOs may better capture the unique heterogeneity and functionality of age fibroblasts compared to hiPSC-derived fibroblasts since they are isolated from adult human myocardium and may consist of a more diverse subpopulation of fibroblasts. Furthermore, maturing or aging iPSC-derived cardiac cell types for future isogenic cardiac microtissues may be necessary to create higher fidelity cardiovascular disease models.

In summary, this study highlights the strengths and limitations of in vitro cardiovascular models for disease modeling and drug discovery. Our study shows that a 3D cell culture environment fosters tissue formation. Including other primary cell types within our hCOs enhanced the transcriptomic similarity to the complex cellular and functional dynamics of human myocardium. Further, our study suggests that future studies should focus on improving the heterogeneity and maturity of the incorporated cell populations to create a more biomimetic model of human myocardium.

## 4. Materials and Methods

### 4.1. D hiPSC-CM Fabrication

Cardiac 3D hiPSC-CMs were fabricated using previously described methods [14]. Briefly, hiPSC-CM (iCell Cardiomyocytes, donor 01434; Fujifilm Cellular Dynamics, Inc.; Madison, WI, USA) were seeded onto 0.1% gelatin-coated 6-well plates (EMD Millipore, Burlington, MA, USA) in iCell Plating Medium. After 2 days in culture, plating media was replaced with iCell Cardiomyocyte Maintenance Media. After 2 more days in culture, cells were detached using TrypLE (Gibco ThermoFisher Scientific; Waltham, MA, USA) for 40 min at 37 °C. We have previously optimized the duration of TrypLE treatment to maximize cardiomyocyte detachment while maintaining viability (data not shown). Cells were seeded into 2% agarose molds casted from silicon mastermolds (Microtissues, Inc.; Sharon, MA, USA). Each agarose mold consists of 35 micromold recesses that allow suspended cells to settle into and encourage cell–cell adhesion to form 3D hiPSC-CM microtissues of approximately 200 microns in diameter. For 14 days, 3D hiPSC-CMs were maintained in culture in Maintenance Media, replacing media every 2 days.

### 4.2. RNA-Sequencing of 3D hiPSC-CM and hCOs

RNA-seq was performed on 3D hiPSC-CM using previously described methods [12]. Briefly, total RNA was isolated from 35 3D hiPSC-CM spheroids and hCOs per replicate (i.e., one agarose mold per replicate), 14 days after fabrication using the Omega bio-tek E.Z.N.A Total RNA kit with the addition of the homogenizer columns (Omega bio-tek, Inc.; Norcross, GA, USA). RNA-seq libraries were prepped using the TruSeq RNA Sample Prep Kit (Illumina, Inc.; San Diego, CA, USA) using 100–200 ng of RNA, according to the manufacturer’s protocol. FastQ files were generated after performing RNA-seq using an Illumina HiSeq2500 (Illumina, Inc.; San Diego, CA, USA) with each mRNA library sequenced to a depth of ~50 million reads. A single-end 50-cycle sequencing protocol was used, followed by data processing using the Illumina quality control procedures. RNA-seq of hCOs was performed previously [12] and is publicly available within the NCBI GEO database, under the accession number GSE113871.

### 4.3. D hiPSC-CMs and Myocardium FASTQ Data Collection

To reduce variability between iPSC-CM samples, RNA-sequencing data were obtained from a publicly available dataset (GSE91383, SRP094851 [13]) with iCell cardiomyocytes matching the iPSC-CM cell line used in our 3D hiPSC-CMs and hCOs samples. In Necela et al. [13], iPSC-CM were cultured onto fibronectin (5 mg/mL) coated wells and cultured for 12 days according the manufacturer’s instructions (Fujifilm Cellular Dynamics, Inc., Madison, WI, USA) prior to RNA isolation for RNA-sequencing. FASTQ files for non-treated iCell iPSC-CM, healthy adult and fetal, human myocardium and an additional human adult ventricle sample were obtained from the European Nucleotide Archive under the study numbers SRP094851 [13], SRP049449 [24] and SRP097153 [25], respectively.

### 4.4. Genome Alignment and Gene Counts Generation

Genome alignment was performed using the toolset HOMER (v4.11) [43] for each FASTQ file per sample. Genome alignment was performed using STAR (v2.7.0) [44] to the GRCh37(hg19) reference genome, and subsequent raw gene counts were generated using the RNA-seq toolset from HOMER.

### 4.5. Principal Component Analysis and Gene Set Enrichment Analysis

Principal component analysis (PCA) and the subsequent gene set enrichment analysis (GSEA) was performed as previous reported [12] using the Bioconductor (v3.1.2) [45,46] package in RStudio (v1.3.1093) (R language v3.6.1). Gene counts matrix was loaded into RStudio and genes containing no counts or single counts were removed. The package “DESeq2” (v1.32) [47] was used to create a summarized experiment object. For PCA analysis, an r-log transformation was performed on the summarized experiment object to stabilize the variation and the subsequent PCA analysis was performed using the “prcomp” function. PCA plots were generated using the package “ggplot2” (v3.3.5) [48]. Gene loadings for PC1 and PC2 were used as gene rankings for GSEA (v4.0.3) (Broad Institute [49,50]). GSEA was performed for the biological pathways gene ontology database, C5.bp.v7.0.symbols.gmt (19 May 2020). Gene ontology (GO) terms were considered significant if they exhibited a *p*-value less than 0.01 and a false discovery rate (FDR) of less than 0.25. GO terms were then loaded into REViGO (http://revigo.irb.hr/; 19 May 2020) [15] alongside their normalized enrichment score (NES) to remove redundant GO terms.

### 4.6. Differential Gene Expression Analysis and Metascape Pathway Analysis

The R package, “DESeq2”, was used to perform the differential gene expression (DGE) analysis [47]. Briefly, a gene count matrix containing cardiac organoid, 3D hiPSC-CM and 2D hiPSC-CM samples (*n* = 3 per group) were loaded into RStudio. The following two DEG analyses were performed using DESeq2: (1) hCOs compared to 2D hiPSC-CMs and (2) 3D hiPSC-CMs compared to 2D hiPSC-CMs. DEG’s that exhibited a log2fold change greater than 1.33 (Fold Change > 2.5) or less than 1.33 (Fold change < −2.5) and an adjusted *p*-value < 0.001 were considered in our study. The DEG datasets were further split into upregulated (log2fold > 1.33) and downregulated (log2fold change < 1.33) gene lists. Both, the upregulated and downregulated gene set were loaded into a Venn diagram webtool, (http://bioinformatics.psb.ugent.be/webtools/Venn/; 18 August 2020), to isolate uniquely and similarly differential expressed genes in hCOs and 3D hiPSC-CMs. The resulting gene lists corresponding to DGE in both hCOs and 3D hiPSC-CM were loaded into Metascape [23] (18 August 2020) for pathway analysis. A custom analysis was performed for enrichment of pathways in the gene ontology database.

### 4.7. K-Means Cluster Analysis of Cardiomyocyte Specific Gene Set

A custom list of cardiomyocyte specific genes was created based on previous reports [33,34,35]. Genes were categorized by known functions associated with cardiomyocytes (e.g., calcium handling, other ion transport, metabolism, sarcomere and transcription factor). We created count matrices consisting of samples for the following 2 comparisons: (1) 2D hiPSC-CMs, 3D hiPSC-CMs, hCOs, fetal and adult ventricles and (2) 2D hiPSC-CMs, 3D hiPSC-CMs and hCOs. The rlog transformed data were reduced to the genes composing the cardiomyocyte specific gene set. Expression values were scaled using z-score across samples per gene using the RStudio’s scale function. We performed a k-means cluster analysis using the RStudio package “stats” (v4.1). We first calculated the optimal number of clusters for each matrix by extracting the maximum average silhouette width using the R package “cluster” (v3.2). We then performed k-means cluster analysis using the optimal cluster for each normalized count matrices. We plotted the k-means cluster analysis using the “pheatmap” (v1.0.12) package with hierarchical clustering of the dataset of 2D hiPSC-CMs, 3D hiPSC-CMs, hCOs, fetal and adult ventricles using Euclidean distance.

### 4.8. Gene Set Variation Analysis

Cell-type specific gene sets were obtained from the publicly available Heart Cell Atlas [30]. We defined the following cell groups to separate cell-type specific genes: (1) ventricular cardiomyocytes, (2) atrial cardiomyocytes, (3) ventricular fibroblasts, (4) endothelial cells, (5) adipocytes, (6) pericytes, (7) smooth muscle cells, (8) neural cells and (9) immune cells. The cardiac cell type gene sets were derived from supplementary tables 6 (cardiomyocytes), 8 (vascular cells), 11 (fibroblasts), 14 (immune cells), 20 (neuronal cells) and 21 (adipocytes) from the Human Heart Cell Atlas. The 50 highest expressed genes per subcluster were combined into one of our defined gene sets based on cell type and replicate genes were removed (i.e., the 50 highest expressed genes within each 13 subclusters of fibroblasts were combined to create the “Fibroblast” gene set). Raw gene counts of all experimental groups were normalized to log_2_(counts per million) using the R package “EdgeR” (v3.34). GSVA was, subsequently, performed using the “gsva” R package (v1.40.1) [51,52,53]. 

## Data Availability

The 3D hiPSC-CM RNA-seq data has been submitted to Genome Expression Omnibus (GEO; National Institute of Health; Bethesda, Maryland, USA) under the accession number GSE181397.

## Supplementary Materials

The following are available online at https://www.mdpi.com/article/10.3390/ijms22168482/s1, Table S1: Comprehensive list of GO terms corresponding to PC1 and PC2 from GSEA of in vitro cardiac models. Table S2: Gene lists of unique and similar, differentially expressed genes in 3D hiPSC-CM and hCOs. Table S3: Comprehensive list of GO terms corresponding to PC1 and PC2 from GSEA of in vitro cardiac models, Table S4: Gene lists of cell-types derived from the Human Heart Atlas. Table S5: Cardiomyocyte specific gene set.
